# *N*-[4-(Azetidin-1-ylsulfon­yl)phen­yl]-*N*-(2,4-difluoro­benz­yl)acetamide

**DOI:** 10.1107/S1600536812038342

**Published:** 2012-09-15

**Authors:** Jian-Mei Lin, Jia-Wen Li, Jing-Song Lv

**Affiliations:** a Sichuan Provincial People’s Hospital, Chengdu 610072, People’s Republic of China; b Laboratory of Bioorganic & Medicinal Chemistry, School of Chemistry and Chemical Engineering, Southwest University, Chongqing 400715, People’s Republic of China; c School of Chemistry and Chemical Engineering, Bijie University, Bijie, Guizhou 551700, People’s Republic of China

## Abstract

In the title mol­ecule, C_18_H_18_F_2_N_2_O_3_S, the dihedral angle between the benzene rings is 79.40 (11)°. The 2,4-difluoro­benzyl and azetidine fragments adopt a *trans* arrangement relative to the central benzene ring. In the crystal, weak C—H⋯O hydrogen bonds connect mol­ecules into a two-dimensional network parallel to (001).

## Related literature

For the pharmacological activity of sulfonamides, see: Song *et al.* (2007 ▶); Wang, Wang *et al.* (2010 ▶); Wang, Wan & Zhou (2010 ▶); Wang, Gan *et al.* (2010 ▶).
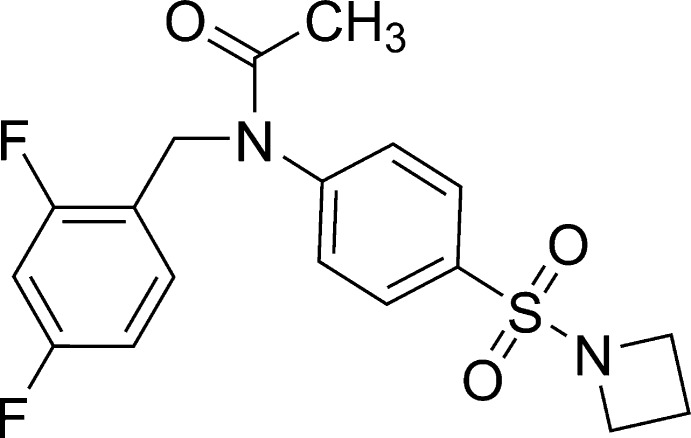


## Experimental

### 

#### Crystal data

C_18_H_18_F_2_N_2_O_3_S
*M* *_r_* = 380.40
Monoclinic, 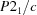

*a* = 8.7793 (15) Å
*b* = 8.4442 (15) Å
*c* = 23.810 (4) Å
β = 97.312 (6)°
*V* = 1750.8 (5) Å^3^
*Z* = 4
Mo *K*α radiation
μ = 0.23 mm^−1^
*T* = 293 K
0.22 × 0.21 × 0.20 mm

#### Data collection

Bruker SMART CCD diffractometer
Absorption correction: multi-scan (*SADABS*; Bruker, 2001 ▶) *T*_min_ = 0.000, *T*_max_ = 0.001
14668 measured reflections
3077 independent reflections
2684 reflections with *I* > 2σ(*I*)
*R*_int_ = 0.036

#### Refinement

*R*[*F* ^2^ > 2σ(*F* ^2^)] = 0.047
*wR*(*F* ^2^) = 0.143
*S* = 1.04
3077 reflections
236 parameters
H-atom parameters constrained
Δρ_max_ = 0.34 e Å^−3^
Δρ_min_ = −0.38 e Å^−3^

### 

Data collection: *SMART* (Bruker, 2001 ▶); cell refinement: *SAINT* (Bruker, 2001 ▶); data reduction: *SAINT*; program(s) used to solve structure: *SHELXS97* (Sheldrick, 2008 ▶); program(s) used to refine structure: *SHELXL97* (Sheldrick, 2008 ▶); molecular graphics: *SHELXTL* (Sheldrick, 2008 ▶); software used to prepare material for publication: *SHELXTL*.

## Supplementary Material

Crystal structure: contains datablock(s) global, I. DOI: 10.1107/S1600536812038342/lh5527sup1.cif

Structure factors: contains datablock(s) I. DOI: 10.1107/S1600536812038342/lh5527Isup2.hkl

Additional supplementary materials:  crystallographic information; 3D view; checkCIF report

## Figures and Tables

**Table 1:** Hydrogen-bond geometry (Å, °)

*D*—H⋯*A*	*D*—H	H⋯*A*	*D*⋯*A*	*D*—H⋯*A*
C5—H3⋯O3^i^	0.93	2.44	3.336 (3)	162
C11—H9⋯O1^ii^	0.93	2.51	3.406 (3)	162
C17—H18*B*⋯O1^iii^	0.97	2.56	3.509 (4)	166

Symmetry codes: (i) 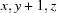; (ii) 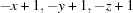; (iii) 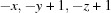.

**Fig. 1.**
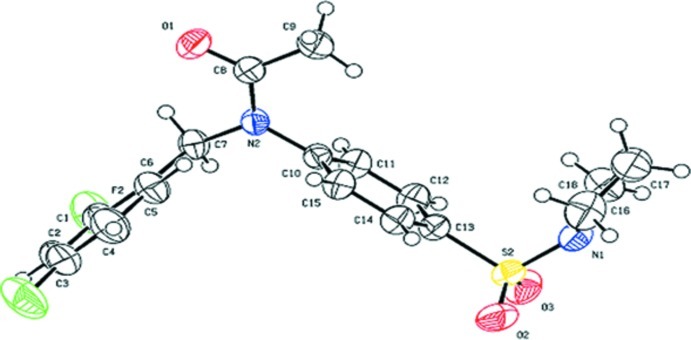
The molecular structure of (I), showing the atom-numbering scheme. Displacement ellipsoids are drawn at the 50% probability level.

**Fig. 2.**
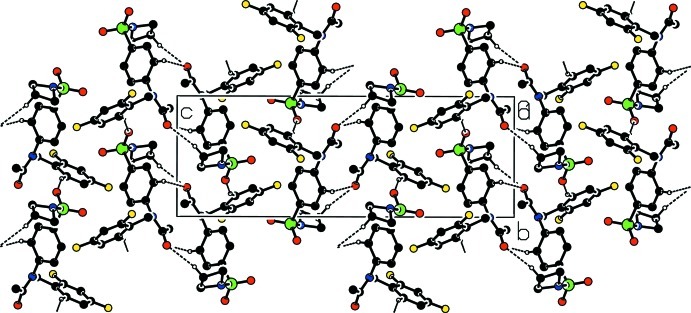
Part of the crystal structure with hydrogen bonds shown as dashed lines.

## supplementary crystallographic information

### Comment

Sulfonamides were extensively employed as effective antimicrobial agents for the prevention and cure of bacterial infections in humanbiological systems as early as 70 years ago, and recently have aroused considerable interest in biology and medicine for their diversified pharmacological activities including antibacterial, antifungal, antiviral, antitumor, anti-inflammatory and as carbonic anhydrase inhibitors (Song, *et al.*, 2007; Wang, Wang *et al.*, 2010). Our interest is to develop novel sulfonamide derivatives as antimicrobial agents and some structrally related sulfonamides have been reported as bioactive agents (Wang, Wan & Zhou, 2010; Wang & Gan *et al.*, 2010). Herein, we report the crystal structure of the title compound (I).

In the molecule of (I) (Fig. 1) the dihedral angle between the two benzene rings is 79.40 (11)°. The 2,4-difluorobenzyl and azetidine fragments adopt a trans arrangement relative to the central benzene ring. In the crystal, weak C—H···O hydrogen bonds connect molecules into a two-dimensional network (Fig. 2) parallel to (001).

### Experimental

A suspension of *N*-(4-(azetidin-1-ylsulfonyl)phenyl)acetamide (0.8 g, 3.0 mmol) and potassium carbonate (0.5 g, 3.6 mmol) was stirred in acetonitrile (30 mL) at 343 K. After half an hour, 1-(bromomethyl)-2,4-difluorobenzene (0.6 g, 3.0 mmol) was added, and the progress of the reaction was monitored by TLC. Upon completion, the reaction was extracted with chloroform (3 × 20 mL). The filtrate was concentrated and then directly purified by chromatographic column (petroleum ether/ethyl acetate) to afford the title compound (I). A crystal suitable for X-ray analysis was grown from a solution of (I) in a mixture of acetone and ethyl acetate by slow evaporation at room temperature.

### Refinement

H atoms were placed at calculated position with C—H = 0.93 Å (aromatic), 0.97Å (methylene) 0.96 Å (methyl). The *U*_iso_(H) value was set equal to 1.2U_eq_(C) or 1.5U_eq_(C_methyl_).

### Crystal data

**Table:**

C_18_H_18_F_2_N_2_O_3_S	*F*(000) = 792
*M**_r_* = 380.40	*D*_x_ = 1.443 Mg m^−^^3^
Monoclinic, *P*2_1_/*c*	Mo *K*α radiation, λ = 0.71073 Å
Hall symbol: -P 2ybc	Cell parameters from 2005 reflections
*a* = 8.7793 (15) Å	θ = 1.3–25.0°
*b* = 8.4442 (15) Å	µ = 0.23 mm^−^^1^
*c* = 23.810 (4) Å	*T* = 293 K
β = 97.312 (6)°	Plate, colourless
*V* = 1750.8 (5) Å^3^	0.22 × 0.21 × 0.20 mm
*Z* = 4	

### Data collection

**Table:**

Bruker SMART CCD diffractometer	3077 independent reflections
Radiation source: fine-focus sealed tube	2684 reflections with *I* > 2σ(*I*)
Graphite monochromator	*R*_int_ = 0.036
φ and ω scans	θ_max_ = 25.0°, θ_min_ = 1.7°
Absorption correction: multi-scan (*SADABS*; Bruker, 2001)	*h* = −10→10
*T*_min_ = 0.000, *T*_max_ = 0.001	*k* = −9→10
14668 measured reflections	*l* = −25→28

### Refinement

**Table:**

Refinement on *F*^2^	Secondary atom site location: difference Fourier map
Least-squares matrix: full	Hydrogen site location: inferred from neighbouring sites
*R*[*F*^2^ > 2σ(*F*^2^)] = 0.047	H-atom parameters constrained
*wR*(*F*^2^) = 0.143	*w* = 1/[σ^2^(*F*_o_^2^) + (0.0875*P*)^2^ + 0.736*P*] where *P* = (*F*_o_^2^ + 2*F*_c_^2^)/3
*S* = 1.04	(Δ/σ)_max_ = 0.001
3077 reflections	Δρ_max_ = 0.34 e Å^−^^3^
236 parameters	Δρ_min_ = −0.38 e Å^−^^3^
0 restraints	Extinction correction: *SHELXL*, Fc^*^=kFc[1+0.001xFc^2^λ^3^/sin(2θ)]^-1/4^
Primary atom site location: structure-invariant direct methods	Extinction coefficient: 0.017 (2)

### Special details

**Table:**

Geometry. All e.s.d.'s (except the e.s.d. in the dihedral angle between two l.s. planes) are estimated using the full covariance matrix. The cell e.s.d.'s are taken into account individually in the estimation of e.s.d.'s in distances, angles and torsion angles; correlations between e.s.d.'s in cell parameters are only used when they are defined by crystal symmetry. An approximate (isotropic) treatment of cell e.s.d.'s is used for estimating e.s.d.'s involving l.s. planes.
Refinement. Refinement of *F*^2^ against ALL reflections. The weighted *R*-factor *wR* and goodness of fit *S* are based on *F*^2^, conventional *R*-factors *R* are based on *F*, with *F* set to zero for negative *F*^2^. The threshold expression of *F*^2^ > σ(*F*^2^) is used only for calculating *R*-factors(gt) *etc*. and is not relevant to the choice of reflections for refinement. *R*-factors based on *F*^2^ are statistically about twice as large as those based on *F*, and *R*- factors based on ALL data will be even larger.

### Fractional atomic coordinates and isotropic or equivalent isotropic displacement parameters (Å^2^)

**Table:**

	*x*	*y*	*z*	*U*_iso_*/*U*_eq_	
C1	0.6694 (2)	0.5645 (3)	0.33594 (10)	0.0447 (5)	
C6	0.5341 (2)	0.5834 (2)	0.35904 (9)	0.0365 (5)	
C5	0.4212 (3)	0.6715 (3)	0.32755 (10)	0.0490 (6)	
H3	0.3291	0.6899	0.3419	0.059*	
C4	0.4418 (3)	0.7332 (3)	0.27524 (11)	0.0597 (7)	
H4	0.3647	0.7921	0.2544	0.072*	
C3	0.5781 (3)	0.7056 (3)	0.25474 (11)	0.0585 (7)	
C2	0.6955 (3)	0.6231 (3)	0.28413 (12)	0.0580 (7)	
H6	0.7883	0.6073	0.2700	0.070*	
C13	0.1006 (2)	0.1070 (3)	0.36580 (9)	0.0385 (5)	
C12	0.2177 (3)	0.0895 (3)	0.41033 (10)	0.0432 (5)	
H8	0.2401	−0.0099	0.4261	0.052*	
C11	0.3003 (2)	0.2199 (3)	0.43111 (9)	0.0403 (5)	
H9	0.3793	0.2088	0.4608	0.048*	
C10	0.2657 (2)	0.3687 (2)	0.40761 (8)	0.0340 (5)	
C14	0.0659 (2)	0.2550 (3)	0.34245 (9)	0.0416 (5)	
H11	−0.0129	0.2661	0.3127	0.050*	
C15	0.1482 (2)	0.3860 (3)	0.36341 (10)	0.0408 (5)	
H12	0.1249	0.4855	0.3479	0.049*	
C16	−0.2870 (3)	0.0478 (3)	0.36281 (14)	0.0658 (8)	
H13A	−0.3687	0.0261	0.3324	0.079*	
H13B	−0.2526	0.1567	0.3616	0.079*	
C8	0.3049 (2)	0.6238 (2)	0.45767 (9)	0.0370 (5)	
C9	0.1495 (3)	0.6048 (3)	0.47658 (11)	0.0512 (6)	
H15A	0.1050	0.5065	0.4625	0.077*	
H15B	0.0846	0.6909	0.4621	0.077*	
H15C	0.1594	0.6050	0.5172	0.077*	
C7	0.5160 (2)	0.5089 (3)	0.41508 (9)	0.0401 (5)	
H7A	0.5562	0.4019	0.4155	0.048*	
H7B	0.5774	0.5679	0.4448	0.048*	
C18	−0.1690 (3)	−0.0912 (4)	0.42815 (12)	0.0616 (7)	
H17A	−0.0888	−0.0364	0.4524	0.074*	
H17B	−0.1762	−0.2011	0.4394	0.074*	
C17	−0.3228 (3)	−0.0043 (4)	0.42063 (14)	0.0686 (8)	
H18A	−0.4113	−0.0735	0.4195	0.082*	
H18B	−0.3289	0.0821	0.4471	0.082*	
N1	−0.1609 (2)	−0.0694 (2)	0.36736 (9)	0.0457 (5)	
N2	0.35756 (18)	0.5030 (2)	0.42793 (7)	0.0351 (4)	
O1	0.38424 (18)	0.74030 (19)	0.47069 (7)	0.0496 (4)	
O2	−0.0512 (3)	−0.0306 (2)	0.27960 (8)	0.0689 (6)	
O3	0.08117 (19)	−0.19746 (19)	0.35635 (9)	0.0595 (5)	
F1	0.5958 (3)	0.7614 (3)	0.20283 (8)	0.0956 (7)	
F2	0.78369 (17)	0.4820 (2)	0.36687 (8)	0.0728 (5)	
S2	−0.00544 (6)	−0.05936 (7)	0.33804 (2)	0.0439 (2)	

### Atomic displacement parameters (Å^2^)

**Table:**

	*U*^11^	*U*^22^	*U*^33^	*U*^12^	*U*^13^	*U*^23^
C1	0.0383 (11)	0.0416 (13)	0.0557 (14)	−0.0018 (9)	0.0115 (10)	−0.0043 (10)
C6	0.0371 (10)	0.0311 (10)	0.0421 (11)	−0.0042 (8)	0.0083 (9)	−0.0032 (8)
C5	0.0438 (12)	0.0515 (14)	0.0535 (14)	0.0057 (10)	0.0137 (10)	0.0076 (11)
C4	0.0662 (16)	0.0605 (16)	0.0530 (14)	0.0034 (13)	0.0103 (12)	0.0172 (12)
C3	0.0729 (17)	0.0577 (16)	0.0481 (14)	−0.0133 (13)	0.0197 (12)	0.0055 (12)
C2	0.0581 (14)	0.0542 (15)	0.0678 (17)	−0.0120 (12)	0.0321 (13)	−0.0068 (13)
C13	0.0394 (11)	0.0356 (11)	0.0408 (11)	−0.0024 (9)	0.0056 (9)	−0.0033 (9)
C12	0.0475 (12)	0.0318 (11)	0.0494 (13)	0.0023 (9)	0.0024 (10)	0.0060 (9)
C11	0.0396 (10)	0.0384 (12)	0.0411 (11)	0.0002 (9)	−0.0021 (9)	0.0051 (9)
C10	0.0327 (10)	0.0326 (11)	0.0373 (10)	−0.0003 (8)	0.0074 (8)	0.0001 (8)
C14	0.0404 (11)	0.0413 (12)	0.0412 (11)	0.0013 (9)	−0.0021 (9)	0.0025 (9)
C15	0.0404 (11)	0.0346 (11)	0.0463 (12)	0.0024 (9)	0.0013 (9)	0.0055 (9)
C16	0.0453 (13)	0.0574 (17)	0.092 (2)	0.0090 (12)	0.0002 (14)	0.0004 (14)
C8	0.0399 (11)	0.0355 (12)	0.0352 (10)	0.0009 (9)	0.0029 (8)	0.0033 (9)
C9	0.0464 (12)	0.0544 (14)	0.0551 (14)	0.0036 (11)	0.0156 (11)	−0.0045 (11)
C7	0.0310 (10)	0.0428 (12)	0.0466 (12)	0.0017 (8)	0.0056 (9)	0.0045 (9)
C18	0.0587 (15)	0.0660 (17)	0.0618 (16)	0.0032 (13)	0.0146 (13)	0.0028 (13)
C17	0.0558 (15)	0.0649 (18)	0.088 (2)	0.0014 (13)	0.0218 (15)	−0.0165 (16)
N1	0.0413 (10)	0.0402 (11)	0.0541 (12)	−0.0020 (8)	0.0009 (9)	−0.0068 (8)
N2	0.0316 (8)	0.0346 (9)	0.0395 (9)	−0.0028 (7)	0.0068 (7)	0.0008 (7)
O1	0.0548 (9)	0.0397 (9)	0.0534 (10)	−0.0071 (7)	0.0036 (8)	−0.0062 (7)
O2	0.0911 (14)	0.0710 (13)	0.0438 (10)	−0.0216 (11)	0.0058 (9)	−0.0158 (9)
O3	0.0549 (10)	0.0342 (9)	0.0909 (14)	0.0033 (7)	0.0156 (9)	−0.0119 (9)
F1	0.1171 (15)	0.1121 (16)	0.0652 (11)	−0.0096 (12)	0.0409 (11)	0.0288 (10)
F2	0.0422 (8)	0.0833 (12)	0.0961 (13)	0.0171 (8)	0.0213 (8)	0.0172 (9)
S2	0.0484 (4)	0.0368 (4)	0.0468 (4)	−0.0036 (2)	0.0076 (3)	−0.0109 (2)

### Geometric parameters (Å, º)

**Table:**

C1—F2	1.359 (3)	C15—H12	0.9300
C1—C2	1.375 (4)	C16—N1	1.479 (3)
C1—C6	1.381 (3)	C16—C17	1.516 (5)
C6—C5	1.382 (3)	C16—H13A	0.9700
C6—C7	1.502 (3)	C16—H13B	0.9700
C5—C4	1.383 (4)	C8—O1	1.223 (3)
C5—H3	0.9300	C8—N2	1.356 (3)
C4—C3	1.369 (4)	C8—C9	1.499 (3)
C4—H4	0.9300	C9—H15A	0.9600
C3—F1	1.350 (3)	C9—H15B	0.9600
C3—C2	1.362 (4)	C9—H15C	0.9600
C2—H6	0.9300	C7—N2	1.463 (3)
C13—C14	1.386 (3)	C7—H7A	0.9700
C13—C12	1.388 (3)	C7—H7B	0.9700
C13—S2	1.766 (2)	C18—N1	1.470 (3)
C12—C11	1.376 (3)	C18—C17	1.527 (4)
C12—H8	0.9300	C18—H17A	0.9700
C11—C10	1.393 (3)	C18—H17B	0.9700
C11—H9	0.9300	C17—H18A	0.9700
C10—C15	1.384 (3)	C17—H18B	0.9700
C10—N2	1.439 (3)	N1—S2	1.612 (2)
C14—C15	1.380 (3)	O2—S2	1.419 (2)
C14—H11	0.9300	O3—S2	1.4296 (18)
			
F2—C1—C2	118.4 (2)	H13A—C16—H13B	111.1
F2—C1—C6	117.2 (2)	O1—C8—N2	121.09 (19)
C2—C1—C6	124.5 (2)	O1—C8—C9	121.4 (2)
C1—C6—C5	116.1 (2)	N2—C8—C9	117.43 (19)
C1—C6—C7	119.94 (19)	C8—C9—H15A	109.5
C5—C6—C7	123.92 (19)	C8—C9—H15B	109.5
C6—C5—C4	121.6 (2)	H15A—C9—H15B	109.5
C6—C5—H3	119.2	C8—C9—H15C	109.5
C4—C5—H3	119.2	H15A—C9—H15C	109.5
C3—C4—C5	118.5 (2)	H15B—C9—H15C	109.5
C3—C4—H4	120.7	N2—C7—C6	114.27 (17)
C5—C4—H4	120.7	N2—C7—H7A	108.7
F1—C3—C2	118.8 (2)	C6—C7—H7A	108.7
F1—C3—C4	118.3 (3)	N2—C7—H7B	108.7
C2—C3—C4	122.9 (2)	C6—C7—H7B	108.7
C3—C2—C1	116.3 (2)	H7A—C7—H7B	107.6
C3—C2—H6	121.8	N1—C18—C17	88.7 (2)
C1—C2—H6	121.8	N1—C18—H17A	113.9
C14—C13—C12	120.43 (19)	C17—C18—H17A	113.9
C14—C13—S2	119.25 (16)	N1—C18—H17B	113.9
C12—C13—S2	120.32 (17)	C17—C18—H17B	113.9
C11—C12—C13	119.6 (2)	H17A—C18—H17B	111.1
C11—C12—H8	120.2	C16—C17—C18	87.9 (2)
C13—C12—H8	120.2	C16—C17—H18A	114.0
C12—C11—C10	119.96 (19)	C18—C17—H18A	114.0
C12—C11—H9	120.0	C16—C17—H18B	114.0
C10—C11—H9	120.0	C18—C17—H18B	114.0
C15—C10—C11	120.28 (19)	H18A—C17—H18B	111.2
C15—C10—N2	120.25 (18)	C18—N1—C16	91.5 (2)
C11—C10—N2	119.41 (17)	C18—N1—S2	125.64 (16)
C15—C14—C13	119.97 (19)	C16—N1—S2	126.87 (18)
C15—C14—H11	120.0	C8—N2—C10	123.67 (16)
C13—C14—H11	120.0	C8—N2—C7	118.69 (17)
C14—C15—C10	119.72 (19)	C10—N2—C7	117.64 (17)
C14—C15—H12	120.1	O2—S2—O3	120.79 (12)
C10—C15—H12	120.1	O2—S2—N1	106.48 (12)
N1—C16—C17	88.8 (2)	O3—S2—N1	105.75 (11)
N1—C16—H13A	113.8	O2—S2—C13	107.50 (11)
C17—C16—H13A	113.8	O3—S2—C13	107.47 (10)
N1—C16—H13B	113.8	N1—S2—C13	108.35 (10)
C17—C16—H13B	113.8		

### Hydrogen-bond geometry (Å, º)

**Table:**

*D*—H···*A*	*D*—H	H···*A*	*D*···*A*	*D*—H···*A*
C5—H3···O3^i^	0.93	2.44	3.336 (3)	162
C11—H9···O1^ii^	0.93	2.51	3.406 (3)	162
C17—H18*B*···O1^iii^	0.97	2.56	3.509 (4)	166

Symmetry codes: (i) *x*, *y*+1, *z*; (ii) −*x*+1, −*y*+1, −*z*+1; (iii) −*x*, −*y*+1, −*z*+1.
